# Functionalization of Carbon Nanotubes in Polystyrene and Properties of Their Composites: A Review

**DOI:** 10.3390/polym16060770

**Published:** 2024-03-11

**Authors:** Hongfu Li, Guangfei Wang, Ying Wu, Naisheng Jiang, Kangmin Niu

**Affiliations:** School of Materials Science and Engineering, University of Science and Technology Beijing, 30th Xueyuan Road, Haidian District, Beijing 100083, China; wgf863933535@126.com (G.W.); wuying@ustb.edu.cn (Y.W.); naishengjiang@ustb.edu.cn (N.J.)

**Keywords:** CNT/PS composite, functionalization, dispersion, properties

## Abstract

The inherent π–π interfacial interaction between carbon nanotubes (CNTs) and polystyrene (PS) makes the CNT/PS composite a representative thermoplastic nanocomposite. However, the strong van der Waals force among CNTs poses challenges to achieving effective dispersion. This review provides an overview of various CNT functionalization methods for CNT/PS composites, encompassing covalent grafting with PS-related polymers and non-covalent modification. A focus in this section involves the pre-introduction surface modification of CNTs with PS or PS-related polymers, substantially enhancing both CNT dispersibility and interfacial compatibility within the PS matrix. Furthermore, a comprehensive summary of the mechanical, electrical, thermal, and electromagnetic shielding properties of CNT/PS nanocomposites is provided, offering an overall understanding of this material. The surface modification methods of CNTs reviewed in this paper can be extended to carbon material/aromatic polymer composites, assisting researchers in customizing the optimal surface modification methods for CNTs, maximizing their dispersibility, and fully unleashing the various properties of CNTs/polymer composites. Additionally, high-performance CNTs/PS composites prepared using appropriate CNT modification methods have potential applications in areas such as electronic devices, sensors, and energy storage and conversion.

## 1. Introduction

Polystyrene (PS) stands as a widely utilized commercial thermoplastic because of its excellent processing properties, high hardness, colorless transparency, and good chemical inertness. Its applications span diverse fields such as healthcare products, dining (disposable cups and utensils), packaging, and construction materials [1,2,3,4]. With advancing technology, there have been increasing demands for improved characteristics of PS. As a result, there arises a growing demand for enhanced properties in PS, leading to extensive research on PS nanocomposites [5,6,7,8]. Carbon nanotubes (CNTs), owing to their unique physical properties, emerge as key nanofillers capable of endowing PS with superior mechanical, thermal, and electrical attributes, meeting the evolving expectations of researchers.

CNTs, established based on the structure by Iijima in 1991 and mentioned in the literature almost 50 years in advance by scientists from the Soviet Union (USSR) [9,10], present as one-dimensional materials with concentric cylinders formed by the curling of graphene layers. Their nanometer-scale diameter and micrometer-scale length [11,12] classify them into single-wall carbon nanotubes (SWCNTs), double-wall carbon nanotubes (DWCNTs), and multi-wall carbon nanotubes (MWCNTs). CNTs exhibit excellent optical, thermal, electrical, and mechanical properties, providing extensive prospects in nanocomposite materials [13], sensors [14], energy conversion and storage [15], transparent conductors [16], flexible electronics [17], electromagnetic shielding [18], and catalysts [19]. However, the strong van der Waals forces among CNTs result in the formation of bundles or larger agglomerates, making it challenging to disperse them in water, organic solvents, or polymer matrices, limiting their applications in various fields [20,21]. Similarly, achieving proper dispersion of CNTs in PS is a primary challenge in the study of CNTs/PS nanocomposites. Surface modification of CNTs is currently an effective method to enhance their dispersibility, categorized into covalent (acid oxidation, plasma treatment, halogenation, polymer covalent grafting) [22,23,24,25] and non-covalent modifications (π–π interactions, hydrophobic–hydrophilic, electrostatic interactions, hydrogen bonding, non-covalent polymer modification, inorganic nanoparticle modification) [11,26,27,28,29,30]. The modification minimizes or eliminates van der Waals forces, with covalent modification altering the intrinsic properties of CNTs, while non-covalent modification preserves their electronic structure [31,32,33]. Additionally, understanding the degree of covalent grafting modification of CNTs helps in accurately controlling the electromechanical properties of CNTs composites, with methods including thermogravimetric analysis and X-ray photoelectron spectroscopy [34,35].

Existing reviews on the functionalization of CNTs mainly focus on general modification methods [11,31], while they overlook the critical effect of the matrix material in determining the functionalization method. This review addresses this gap, focusing on the surface modification of CNTs in CNTs/PS composites, emphasizing covalent grafting of polymers and non-covalent modification. The term “polymer” predominantly refers to the PS matrix, which can be chemically bonded to CNTs or wrapped/encapsulated through various non-covalent interactions. Utilizing the polymer matrix for CNT modification not only enhances dispersibility but also addresses compatibility issues between CNTs and PS, presenting significant advantages. The review on the functionalization of CNTs for CNT/PS composites also provides valuable insights into the preparation of other carbon material/aromatic polymer composites.

In this review, we provide an overview of the functionalization of CNTs for PS-based composites and their properties. The necessity of this work is elucidated, followed by a detailed exploration of surface modification methods of CNTs in PS, covering both covalent grafting and non-covalent modifications. Additionally, we delve into the impact of incorporating CNTs into PS on mechanical, electrical, and electromagnetic shielding, thermal conductivity, and thermal stability properties. The objective is to offer readers a thorough understanding of CNT surface modification methods in PS and their influence on PS properties, providing insights for future research in carbon-material-reinforced aromatic polymer composites, and expanding the potential application areas of carbon material/aromatic polymer composites.

## 2. General Principles for the Interaction between CNTs and the Polymers

Due to their high surface energy, carbon nanotubes have a propensity to aggregate easily and are prone to encapsulation by polymers during the preparation of composite materials. For instance, the external surface area of a 9 nm diameter bundle comprising 1 nm SWNTs is only 21% of the total surface area of the same number of SWNTs [36]. These resulting interfacial characteristics directly impact the effectiveness of nanotube reinforcements in enhancing the mechanical, thermal, and electrical properties of polymer nanocomposites [37]. To attain the desired properties, it is essential for CNTs to be well dispersed in solvents or polymer solutions. However, achieving controlled dispersion of CNTs in solutions or composite matrices remains challenging due to the strong van der Waals binding energy aggregates associated with the CNTs [38,39]. Understanding the theory underlying the interaction between CNTs and the polymer matrix is crucial for effectively dispersing CNTs and achieving high-performance CNT-modified polymer composite materials.

### 2.1. Types of CNT–Polymer Interactions

Different functionalization states determine the nature of interactions between CNTs and polymers. Untreated CNTs primarily engage in load transfer with the polymer through either van der Waals or π interactions. Alternatively, surface functionalization of CNTs before blending with the polymer is commonly employed to enhance the interaction strength, facilitated by mechanisms like hydrogen and covalent bonding [40]. The associated energies for each interaction mode are detailed in Table 1.

#### 2.1.1. van der Waals and π Interactions

In instances where CNTs lack functionalization and covalent interaction with the polymer, stress transfer occurs via van der Waals or π interactions between the organic material and the 2p orbitals of their graphene-like structure [43]. π interactions resemble electrostatic interactions, wherein the electron-rich π system acts as a region of negative charge capable of interacting with ions, polar molecules, or another π system. Common bonding mechanisms in CNT/polymer systems include π–π stacking, NH–π, and CH–π interactions [44], involving the alignment of a functional group of the polymer with the hexagonal carbon arrangements in the CNT wall. The strength of the chain–CNT interaction hinges on atomic orbital overlap and varies with the polymer’s chemical structure. This is evident in polymers like polystyrene and poly(phenyl acetylene), where aromatic rings in the side chains rotate towards each other rather than the CNT surface. Conversely, polymers with aromatic groups on the backbone, such as poly(p-phenylenevinylene), align their rings parallel to the nanotube surface. Non-covalent π interactions influence chain arrangement along the nanotube surface, leading to larger-scale configurational interplays like bridging [45] and wrapping [46,47,48]. Exploiting π interactions in CNT–polymer composites does not induce defects on the CNT surface and could compromise their stiffness and strength [49,50]. However, π interactions generally provide weak interfacial adhesion, highly reliant on the polymer choice.

#### 2.1.2. Hydrogen Bonding and Covalent Bonding

Hydrogen bonding results from a dipole–dipole interaction between a hydrogen atom from the polymer chain and an electronegative atom (e.g., oxygen, chlorine, or fluorine) grafted onto the CNT surface. Covalent bonding occurs when an atom from the polymer chain and another atom on the CNT surface share an electron pair, forming a direct chemical bond between the CNT and the organic molecule. These bonds involve stronger interaction energies, often replacing weaker pre-existing π interactions between CNTs and the polymer. Numerous studies have employed experimental [51,52] and computational [53,54] methods to elucidate the role of covalent interactions in enhancing the mechanical properties of the composite. Furthermore, individual polymer chains, if sufficiently long, can bond with multiple CNTs, facilitating direct load transfer despite the absence of CNT–CNT contact in the network. Covalent bonding typically requires pre-treatment of CNTs to introduce functional groups that can form chemical bonds with reactive groups on polymer chains. Surface functionalization techniques, such as plasma treatment oxidation [55,56] or reaction with strong acids like sulfuric [57] or nitric acid [58,59], yield carboxyl groups (-COOH) on the CNT sidewalls. However, surface functionalization often involves multiple steps, and the choice of functional group chemistry must align with the polymer system of interest. Alternative approaches that bypass initial CNT surface functionalization efforts are being explored, such as infiltrating the network with a polymer containing chemical groups capable of forming reactive radicals and subsequently bonding covalently with CNTs [51,52]. Covalent chemical functionalization has proven effective in enhancing interface properties, such as interfacial load transfer efficiency, leading to significant improvements in carbon-nanotube-reinforced polymer nanocomposite properties [60,61]. However, this method introduces structural defects into the carbon nanotube surface, thereby compromising their intrinsic properties [62,63]. Consequently, a tradeoff often exists between carbon nanotube and interface properties.

### 2.2. Influence of Functionalization of CNT on Dispersion and CNT–Polymer Interactions

The dispersion of carbon nanotubes in the polymer matrix significantly influences the performance of nanocomposites. Agglomerates and bundles of nanotubes can degrade the mechanical and electrical properties of composites compared to theoretical predictions for individual CNTs [64]. Achieving optimal dispersion presents a challenge, requiring comprehensive studies across different scales, especially for nanocomposites with relatively high nanotube content [37].

Dispersion methods involving intense shear, such as ultrasonication, high-speed stirring, and ball milling, are commonly employed in liquid-phase and powder CNT processing to break down CNTs into shorter segments. Typically, a two-step process is employed, consisting of mixing/sonication followed by stabilization [65]. During the mixing process, mechanical energy is applied to the solution to disperse aggregates, generating localized shear stress through mixer blade rotation or sonication cavitation. This energy input must be controlled to avoid nanotube fracture. After shear stress removal, CNTs re-agglomerate unless stabilized by surfactants, which provide steric hindrance or electrostatic charge repulsion to prevent re-agglomeration [66]. The driving force for re-agglomeration should be less than the van der Waals attraction [67]. Sodium dodecyl sulfate (SDS) is commonly used as a surfactant.

Achieving optimal dispersion of CNTs involves more than addressing geometric considerations related solely to the length and size of the CNTs. It requires a method to isolate individual CNTs from highly entangled and agglomerated structures and subsequently stabilize them within a polymer matrix to prevent further aggregation [68]. Chemical modification of CNT sidewalls is commonly employed to enhance dispersion or solubility in solvents or polymers and to improve interaction and reactivity with polymers via hydrogen bonding [69]. Zeng [70], leveraging Hamaker’s theory, introduced the concept of “weak interactions between carbon nanotubes and polymers”. Surface treatment of CNTs reduces their surface energy and minimizes polymer coating, facilitating the formation of an effective CNT transport network, thus enhancing the thermal and electrical properties of CNT/polyformaldehyde polymer composites and yielding high-performance functional materials. Polymer wrapping around CNTs represents a non-covalent dispersion method. For instance, polymer wrapping of poly(phenylene vinylene) or polystyrene around the CNTs is accomplished through van der Waals interactions and π–π stacking between CNTs and polymer chains containing aromatic rings. This process can lead to the formation of a supramolecular complex of CNTs [71]. Additionally, the endohedral method serves as another non-covalent approach for CNT functionalization, wherein guest atoms or molecules are encapsulated within CNTs through the capillary effect, often occurring at defects on ends or sidewalls. This method includes the insertion of inorganic nanoparticles or biomolecules into CNTs [66,72].

**Figure 1:**
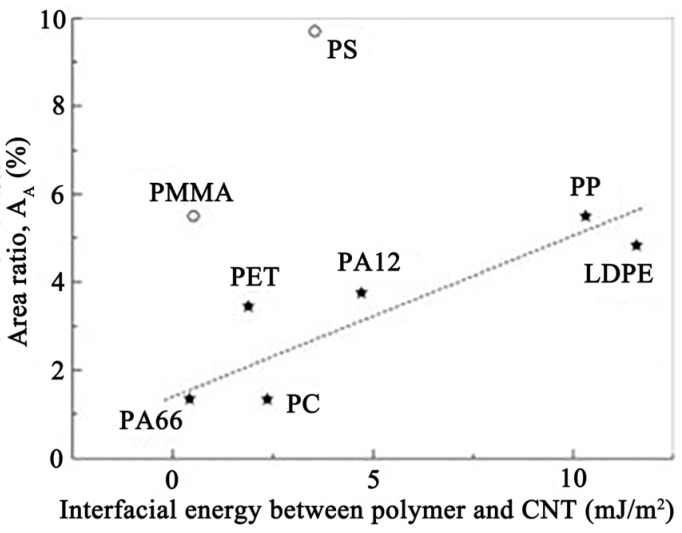
Effect of polymer matrix type on dispersion, area ratio of remaining initial CNT agglomerates *A_A_* versus interfacial energy between nanotubes and polymer (dashed line is for guiding eyes). Reproduced with permission [76]. Copyright © 2011 Woodhead Publishing Limited.

Various factors influencing dispersion have also been explored. Alig et al. [73] reviewed the effects of melt processing conditions and matrix properties on CNT dispersion in polymers, including viscosity, temperature, mixing time, screw speed, and nanotube concentration. Polymer type also significantly impacts dispersion. In Figure 1, the area ratio *A_A_* plotted against the interfacial energy reveals insights into dispersion behavior across different polymers [74]. While there is a general trend of worsening dispersion with increasing interfacial energy, exceptions are observed for polymers such as PS and PMMA, attributed to their chain stiffness and bulky side groups hindering infiltration and interactions with initial agglomerates. Polyolefins, with the highest interfacial energy, exhibit the poorest dispersion, while PA66 shows the best dispersion. A similar trend is observed when plotting the dispersion index against the effective Hamaker constant [75].

### 2.3. Characterization Techniques on CNT–Polymer Interactions

#### 2.3.1. Wetting

Wetting measurements are typically evaluated through parameters such as contact angle, surface tension, and Hamaker constant [37]. Various qualitative and quantitative methods have been employed to analyze the wetting of carbon nanotubes by polymers across different scales [77,78]. Microscopic techniques for wetting assessment often involve drop-on-fiber analysis and utilize optical or electron microscopy to observe contact angles [79]. Additionally, atomic force microscopy (AFM) has been utilized to measure wetting properties using the force balance and Wilhelmy model [80]. In this method, a carbon nanotube attached to an AFM tip is immersed in a liquid polymer bath, where the polymer wets the nanotube and exerts a downward force on the AFM cantilever, which is measured by its deflection. The equilibrium force is expressed as:

(1) $$F_{r}=\gamma_{l}\pi \left(d_{out}cos\theta_{out}+d_{in}cos\theta_{in}\right)\;$$

where *γ_l_* is the surface tension of the liquid, *d_in_* and *d_out_* are the inside and outside diameters of the nanotube, and *θ_in_* and *θ_out_* are the inside and outside contact angles of the nanotube, respectively. It should be mentioned that, in the case of closed nanotubes, or when the polymer does not enter the nanotube, the second term in the bracket is zero. By knowing the surface tension of the liquid and measuring the restoring force, *F_r_*, the contact angle is determined. Dynamic wetting measurements may also involve the retraction of carbon nanotubes from the polymer surface [74].

#### 2.3.2. Spectroscopy

Spectroscopy techniques, including Raman and Fourier transform infrared spectroscopy (FTIR), as well as X-ray scattering, are powerful tools for characterizing nanocomposite materials [81]. These techniques are not only utilized to detect functionalization but also to differentiate between various types of functionalization. Spectroscopy methods can effectively characterize different interaction types, such as van der Waals forces between nanotubes within a bundle, hydrogen bonding, and covalent bonds between nanotubes and the surrounding matrix [37]. Additionally, spectroscopy techniques can detect stress fields in the nanocomposite induced by factors like thermal residual stress or external loads, which manifest as changes in FTIR spectra [82,83]. Raman spectroscopy is particularly useful for evaluating changes in CNT alignment and CNT–polymer load transfer during composite straining, often indicated by shifts in the D- and G-band peaks [84,85]. A significant band shift with increasing strain typically suggests a homogeneous composite morphology with strong interfacial adhesion between CNTs and the polymer. However, assessing the mechanical performance of reinforcement, such as the load transfer limit, necessitates in situ spectroscopy during mechanical testing to indirectly determine the stress transfer limit.

#### 2.3.3. Atomic Force Microscopy—AFM

AFM is one of the most common techniques for interaction measurements. Two different approaches have been taken to employ AFM for interaction measurement studies in carbon nanotube nanocomposites: (a) attach a nanotube to the AFM tip and use a polymer substrate [86], and (b) coat the AFM tip with polymer and use a nanotube substrate [87]. Interaction investigations can be implemented by pullout [88] and peeling experiments [89]. Once the pullout or peeling force is measured, the interfacial shear strength is calculated based on the diameter and the embedded length of the CNT. For instance, Rahmat [81] introduced an interaction measurement parameter called interaction stress by using AFM. The interaction stress is independent of geometry and only depends on the material pair and the environment. It can be used to calculate several interaction parameters, such as interaction force, interaction energy, and internal stress in the interactive bodies. The interaction stress as a function of separation distance was presented, and the validity was examined by using the Hamaker constant [39], which is the van der Waals force scaling constant and can be calculated by various techniques [90]. In In another example, in a recent study, Cai [91] utilized single-molecule force spectroscopy to successfully detect van der Waals forces between individual polymer repeat units and a solid surface in a high-vacuum environment. When the polymer chains, adopting a lying-down conformation, were lifted from the surface, their repeat units sequentially detached with detachment forces ranging from 21 to 54 pN. The force values depend on both the properties of the solid surface and the size of the polymer repeat units. For solid surfaces with a larger Hamaker constant, the van der Waals potential between the molecule and the surface is greater, resulting in stronger van der Waals forces. Similarly, for larger polymer repeat units, the van der Waals forces between the molecule and the surface are stronger due to the additive nature of van der Waals forces.

## 3. Functionalization of CNTs for CNT/PS Composites

### 3.1. Covalent Grafting Using PS-Related Polymers

#### 3.1.1. Atom Transfer Radical Polymerization (ATRP)

**Figure 2:**
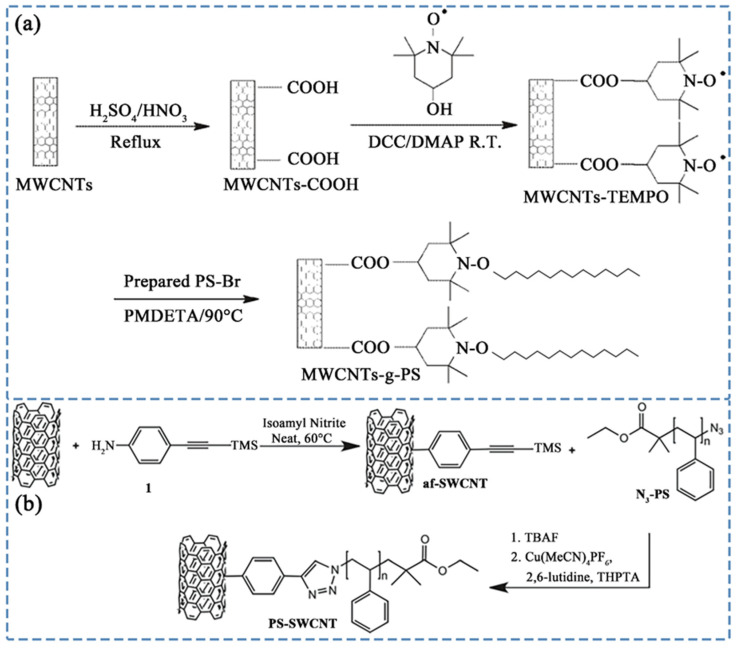
Typical functionalization of CNTs via ATRP: (**a**) Synthesis of MWCNT-g-PS. Reproduced with permission [92]. Copyright 2009, Wiley Periodicals, LLC; (**b**) Synthesis route of PS-SWCNT. Reproduced with permission [94]. Copyright 2013, Wiley-VCH.

In polymer covalent grafting modification, one of the most widely employed and effective methods is ATRP. The ATRP is a controlled radical polymerization technique, which enables the precise synthesis of polymers with well-defined molecular weights and low polydispersity. It involves the reversible activation and deactivation of polymer chains using a transition metal catalyst, including processes of initiation, propagation, deactivation, and termination steps. Sui et al. [92] utilized carboxyl-functionalized CNTs and hydroxyl-functionalized 2,2,6,6-tetramethylpiperidine-1-oxy (TEMPO), successfully obtained MWCNT-TEMPO through esterification. Subsequently, the reaction with brominated PS, prepared via ATRP in the presence of a catalyst, led to the generation of PS covalently grafted MWCNT (MWCNT-g-PS), as shown in Figure 2a. Notably, the study revealed that MWCNT-g-PS demonstrates excellent solubility in organic solvents such as toluene, tetrahydrofuran, and dichloromethane. Liu et al. [93] employed the ATRP method to synthesize PS, followed by the preparation of PS-N_3_ using sodium azide (NaN_3_). Through further covalent grafting of PS-N_3_ and MWCNTs, they successfully obtained the MWCNT-g-PS material. Interestingly, they observed that MWCNT-g-PS, when exposed to selective solvents (PS-philic solvent, MWCNT-phobic solvent), undergoes self-assembly, forming a three-dimensional bundled structure driven by solvent affinity. These assemblies consist of numerous parallel-oriented modified CNTs with loose tails and relatively tight heads. In contrast, unmodified MWCNTs do not exhibit self-assembly behavior in selective solvents. Chadwick et al. [94] synthesized PS covalently grafted CNTs utilizing the “grafting to” method. Through Pschorr-type diazotization, they synthesized af-SWCNT, employed ATRP and substitution reactions to prepare N_3_-PS, and covalently bonded af-SWCNT with N_3_-PS to obtain PS-modified SWCNT (PS-SWCNT), as illustrated in Figure 2b. The study investigated the relationship between molecular weight and solubility of grafted PS [95], revealing that the area occupied by PS chains on CNTs’ surface is proportional to the square of the chain size. Understanding the spatial hindrance of PS chains aids in estimating the barrier size preventing mutual aggregation of CNTs, with the barrier size influencing solubility. The study found that solubility is maximized with moderate molecular weight of PS and relatively high grafting density, emphasizing the role of grafting density and molecular weight in adjusting CNTs’ solubility.

After functionalizing the surface of CNTs, grafting with styrene monomers is a common method for attaching PS to CNTs. Nayak [96] initially prepared SWCNT-COCl through processes such as acid oxidation and thionyl chloride halogenation. Subsequently, styrene monomers were covalently attached to the surface of SWCNT-COCl, and then, using benzoyl peroxide as an initiator, ATRP was employed to obtain PS-grafted SWCNTs. Thermal gravimetric analysis, nuclear magnetic resonance, and infrared spectroscopy confirmed the successful grafting of PS onto the surface of SWCNTs. Additionally, the glass transition temperature (*T_g_*) of pure PS was lower than that of grafted PS. Covalently grafted CNTs exhibited good dispersibility in solvents, and the electrical resistance of the PS composite material prepared with SWCNT-PS was lower than that of pure SWCNT. Furthermore, Lin et al. [97] successfully covalently grafted MWCNTs on the surface of CNTs through multistep chemical modifications. Initially, CNTs prepared by chemical vapor deposition were acid-oxidized to introduce carboxyl groups. Subsequently, they reacted with 4-vinylbenzyl chloride for esterification, resulting in MWCNTs with surface ester groups (MWCNTs-COOR). Finally, under the initiation of azobisisobutyronitrile (AIBN), MWCNTs-COOR underwent covalent grafting with styrene monomers to achieve CNTs with modified PS on the surface. Compared to the original CNTs, the covalent functionalization of PS enhances the compatibility of CNTs within PS. Moreover, due to the spatial hindrance provided by PS, it prevents the aggregation of CNTs, improving the dispersibility of CNTs in PS, as well as the mechanical properties of PS. Additionally, Yang et al. [98] conducted a series of treatments on MWCNTs, including acid oxidation, acylation, ethynylation, and finally covalent grafting of PS with styrene through free radical polymerization to obtain MWCNTs grafted with PS having a brush-rod structure (PS-g-MWCNTs). Furthermore, the study employed de-functionalization to quantitatively investigate the molecular weight and composition of PS in PS-g-MWCNTs. A uniform PS film formed on the surface of CNTs, and the *T_g_* and thermal stability of PS in PS-g-MWCNTs were significantly improved. Moreover, PS-g-MWCNTs exhibited good solubility in solvents and good dispersion in PS, showing some self-assembly characteristics during the evaporation process of the PS-g-MWCNT solution in tetrahydrofuran, forming a nanoneedle structure on a glass substrate. From the above research, it is evident that covalently grafting PS on the surface of CNTs contributes to improving the solubility of CNTs in organic solvents. However, the factors influencing solubility are not clear. Similarly, Hua et al. [99], based on the synthesis of styrene covalently grafted modified MWCNTs (p-MWCNTs), used in situ free radical polymerization to prepare styrene covalently functionalized MWCNTs/PS (p-MWCNTs/PS) composite materials. The study indicated better compatibility between p-MWCNTs and PS, leading to improved dispersibility in PS. Melt blending is one of the commonly used methods for preparing polymer materials, and it can also induce the covalent grafting of CNTs and PS during the melt mixing process. Zhang et al. [100] investigated the impact of melt blending on the interaction between PS and CNTs. The study found that although CNTs and PS have some interaction in solution, this interaction is not sufficient to dissolve CNTs in organic solvents. Notably, CNTs subjected to melt blending can dissolve in organic solvents, and even ultrasonication does not alter their solubility, indicating that melt blending significantly enhances the interaction between CNTs and PS. Research has demonstrated that the mechanism behind the enhanced interaction between CNTs and PS through melt blending is primarily twofold: on one hand, under the combined action of heating and strong shear force, PS molecular chains are prone to degradation, producing large molecular radicals. On the other hand, similar to C_60_, CNTs act as effective radical receptors, leading to chemical covalent grafting interactions between CNTs and large PS radicals. Additionally, CNTs can also undergo π–π stacking physical interactions with the aromatic rings in PS.

PS, synthesized via emulsion polymerization, can be effectively combined with CNTs through covalent grafting. Hu et al. [101] demonstrated covalent bonding modification of CNTs using PS microspheres. The process involved the preparation of PS microspheres through emulsion polymerization, followed by surface modification using benzoyl peroxide. PS-MWCNTs were then obtained through annealing at a specific temperature. Characterization revealed predominant covalent grafting of MWCNTs onto the sidewalls, showcasing high solubility in xylene and toluene solutions. Furthermore, a notable enhancement in the *T_g_* and electrical conductivity of PS was observed.

Supramolecular anisotropic self-assembly stands out as a relatively effective method for CNT surface modification, particularly favorable for CNT dispersion compared to in situ free radical polymerization modification with styrene monomers on the CNT surface. Oliveira et al. [102] presented three methods for MWCNT modification. The first method involved covalently introducing vinyl into hydroxylated MWCNT to obtain MWCNT-O-TMSPMA (3-(trimethoxysilyl)propyl methacrylate), followed by covalent grafting of PS onto MWCNT-O-TMSPMA using free radical polymerization—a process referred to as anisotropic supramolecular self-assembly. The second and third methods utilized AIBN and styrene for in situ free radical polymerization, resulting in MWCNT-COOH@PS and MWCNT@PS materials, respectively. Additionally, a strategy for preparing MWCNT/PS polymer composite materials was proposed, involving the extrusion of modified MWCNTs into masterbatches, which were then mixed with an appropriate number of PS to obtain PS composite materials with varying MWCNT contents. Comparative studies revealed that MWCNT modification through anisotropic supramolecular self-assembly led to superior dispersion, higher elastic modulus, and notable improvements in conductivity and electromagnetic interference shielding compared to the other two modification methods.

In addition to the method involving the grafting of PS onto CNTs, an alternative approach entails covalently grafting or polymerizing other polymers onto PS, followed by the formation of a covalent bond with CNTs. Abbasian et al. [103] achieved hydroxylated polyphenylene (PP) through maleic anhydride grafting and ring-opening polymerization with ethanol amine. They then synthesized a large molecular initiator (PP-Cl) using α-phenyl chloroacetyl chloride and utilized ATRP to react styrene (St) with PP, yielding the PP-g-PSt copolymer. By employing chlorine-terminated PP-g-PSt, they facilitated chemical bonding between carboxylated CNTs and PP-g-PSt under the catalytic action of CuBr, resulting in the formation of the MWCNTs-g-(PP-g-PSt) composite material. The covalent attachment of PP-g-PSt onto the CNTs’ surface enhances the dispersion of CNTs within the polymer matrix and improves their compatibility with the polymer, especially with PS and PP. Zhang et al. [104] initiated the process by preparing poly(acryloyl chloride)-co-polystyrene through free radical polymerization using PS and poly(acryloyl chloride). They subsequently synthesized azide-poly(styrene-co-acrylic acid) using NaN_3_ and reacted it with sp^2^ carbon in MWCNTs under specific conditions in a solution of N, N-dimethylacetamide, resulting in PS covalently grafted modified MWCNTs (MWCNTs@PS). Finally, an in situ suspension polymerization method was employed to obtain MWCNTs@PS/PS microspheres, showcasing antistatic properties suitable for 3D printing technology. These covalent modification approaches provide a viable pathway for expanding into the realm of CNT-reinforced blends/copolymers.

#### 3.1.2. Other Covalent Grafting Methods

**Figure 3:**
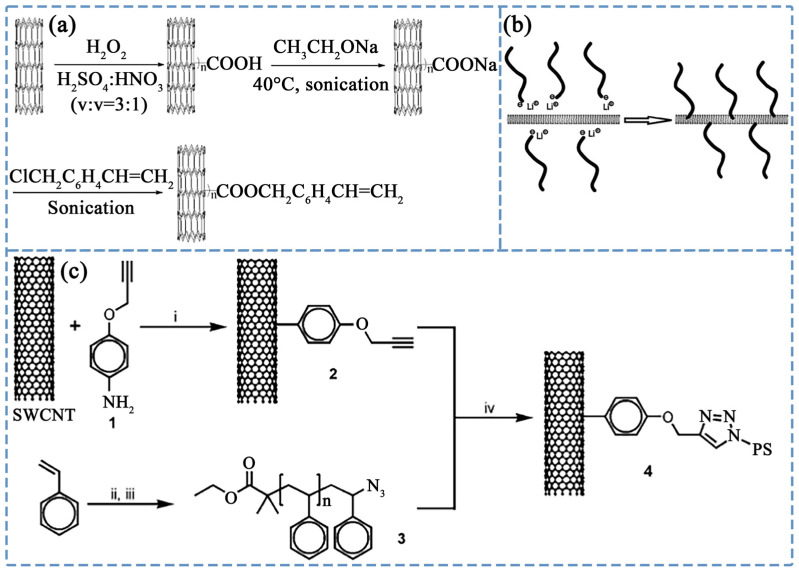
(**a**) A schematic of the styrene-based covalent grafting on MWCNTs. Reproduced with permission [105]. Copyright 2008, Elsevier B.V.; (**b**) An illustration of PS grafting on CNTs using anionic polymerization high-vacuum technique. Reproduced with permission [106]. Copyright 2008, Elsevier B.V.; (**c**) The schematic representation of PS covalent modification of SWCNTs through Huisgen cycloaddition reaction. (i) Isoamyl nitrite, 60 °C; (ii) ethyl 2-bromoisobutyrate, CuBr/2,2′-bipyridine, Dimethylformamide (DMF), 110 °C; (iii) NaN_3_, DMF, room temperature; (iv) Cu(I), DMF. Reproduced with permission [107]. Copyright 2005, American Chemical Society.

In addition to ATRP, various other reactions, such as esterification, anionic polymerization, and Huisgen cycloaddition, have been explored for this purpose. Chen et al. [105] developed styrene-based covalently grafted MWCNTs using a multistep process. Initially, CNTs were oxidized to introduce carboxyl groups, followed by the conversion to carboxylate MWCNTs-COONa. Subsequent esterification with 4-vinylbenzyl chloride resulted in the formation of styrene-based covalently grafted MWCNTs, as illustrated in Figure 3a. The study demonstrated that, in comparison to carboxylation-modified CNTs, styrene-based covalently grafted MWCNTs exhibited superior dispersion in PS. Mountrichas et al. [106] employed anionic polymerization under high vacuum to graft active PS onto the CNT surface, as depicted in Figure 3b. Li et al. [107] utilized Huisgen cycloaddition to graft alkynyl-functionalized single-walled CNTs (SWCNTs) with azide-functionalized PS polymer, shown in Figure 3c. The mild reaction conditions and high grafting density resulted in polymer-functionalized SWCNTs with excellent solubility in various solvents.

Covalent grafting modification of CNTs not only enhances their solubility in solution and dispersibility in polymers but also improves compatibility with the PS matrix. This improved compatibility enhances interfacial interactions, facilitating efficient load transfer and significantly enhancing the electromechanical properties of the polymer material. However, it is crucial to note that covalent polymer modification introduces defects on the sidewalls of CNTs, potentially compromising their intrinsic properties. Therefore, a careful balance must be considered between the inherent characteristics of CNTs and the strength of the interface binding when conducting covalent polymer modification.

### 3.2. Non-Covalent Modification Using PS-Related Polymers

Non-covalent polymer modification mainly refers to polymer wrapping or absorption, where the polymer forms a coating around the CNTs. By creating a polymer coating, spatial hindrance is provided, effectively preventing the aggregation of CNTs [108]. Polymer wrapping alters the surface properties of CNTs, promoting better compatibility with polymers. The advantage of non-covalent polymer modification is that it does not alter the structure of CNTs, thus preserving their mechanical properties. However, non-covalent polymer modification has the drawback of weaker interactions between the modified polymer and CNTs, which may result in a relatively lower efficiency of load transfer.

**Figure 4:**
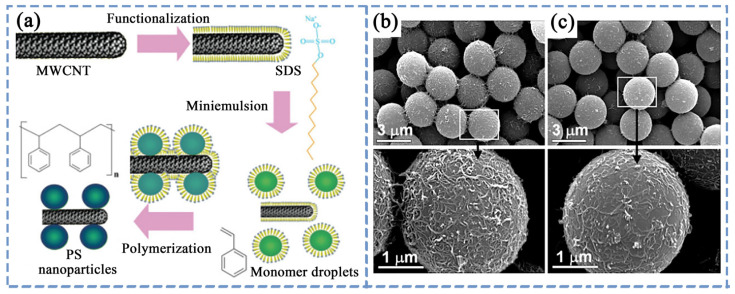
(**a**) The schematic representation of MWCNTs with PS nanoparticles adsorbed on the surface prepared by in-situ microemulsion polymerization. Reproduced with permission [111]. Copyright 2009, Wiley Periodicals, Inc.; (**b**,**c**) the adsorption on the surface of PS microspheres after modifying CNTs with CTAB and NaDDBS, respectively. Reproduced with permission [113]. Copyright 2005, American Chemical Society.

In PS, utilizing π–π stacking interactions to achieve non-covalent modification of CNTs is the most common method. Zhao et al. [109] studied the impact of PS-modified CNTs on the dispersion properties of CNTs through solution blending, relying on non-covalent interactions between CNTs and PS. The research found that in solution, PS tends to adsorb on the surface of CNTs. The adsorption of PS helps stabilize CNTs dispersed by ultrasound, leading to the formation of a uniformly dispersed solution. Additionally, after solution washing, the PS attached to the surface of CNTs remains retained. PS-modified CNTs, once dried, can be redispersed into the solvent and form a stable solution again. Moreover, Rivin et al. [110] employed non-covalent physical adsorption to attach a layer of PS less than 2 nm thick to the surface of CNTs, which was then introduced into PS. The study found that CNTs with nanoscale PS adsorption exhibited good dispersion in PS, and mechanical properties were also improved. Similarly, Patole et al. [111] used an in situ microemulsion polymerization method to prepare MWCNTs with PS nanoparticles adsorbed on the surface, as shown in Figure 4a. PS nanoparticles adsorbed on the surface of CNTs through π–π stacking, allowing CNTs to achieve uniform dispersion in organic solvents. Zhang et al. [112] used dodecyl sodium sulfate to assist in dispersing acid-treated CNTs, obtaining a suspension of CNTs in water. This was then mixed with an emulsion of PS prepared using emulsion polymerization under ultrasound, followed by freeze-drying to obtain well-dispersed MWCNTs/PS composite material. The research found that the dispersion of CNTs in the CNTs/PS composite material could be predicted through dynamic rheological behavior. In addition, Jin et al. [113] first treated CNTs with four different surfactants and then introduced PS or poly(methyl methacrylate) (PMMA) into the solution of surfactant-modified CNTs. Surfactants could enhance hydrophobic interactions during interfacial contact. The study found that compared to the anionic sodium dodecyl SDS and non-ionic Triton X-100, the cationic cetyltrimethylammonium bromide (CTAB)- or sodium dodecylbenzenesulfonate (NaDDBS)-modified CNTs exhibited stronger interfacial interactions with PS and PMMA (Figure 4b,c show the adsorption on the surface of PS microspheres after modifying CNTs with CTAB and NaDDBS, respectively). Additionally, the smaller the diameter of CNTs, the greater the adsorption on the surfaces of PMMA and PS.

In addition to the initial dispersion of CNTs, the polymerization method of PS also affects its wrapping on the surface of CNTs. Ham et al. [114] used three methods to prepare PS-modified CNTs: fine emulsion polymerization, emulsion polymerization, and PS latex–SWCNT mixture. The first two emulsion polymerization methods produced PS-wrapped SWCNTs, with the conventional emulsion polymerization method resulting in fully encapsulated CNTs by PS, while the fine emulsion polymerization method yielded CNTs partially covered. Both types of CNTs prepared by emulsion polymerization showed good dispersion in the good solvent for PS. However, when PS latex and SWCNTs were mixed, CNTs precipitated from the solvent. The good dispersion of non-covalently modified CNTs prepared by emulsion polymerization is based on the adsorption of PS on the nanotube surface. The crosslinking agent added during the fine emulsion polymerization process causes PS to adhere better to the nanotube surface, but excessive crosslinking is detrimental to the dissolution of CNTs in the solvent. In addition, experimental results show that modified CNTs composites prepared by fine emulsion polymerization have higher mechanical strength and better conductivity. The exposed area of CNTs increases with the increase in the amount of CNTs used in fine emulsion polymerization, and the conductivity also increases accordingly. The interaction between CNTs and PS, as well as their dispersion in PS, is influenced by the conformational regularity of PS. London et al. [115], through experiments and simulations, demonstrated that compared to random PS, CNTs interact more strongly with isotactic PS. Moreover, when the CNT loading is <0.5%, the dispersion of CNTs in the isotactic PS matrix is better. Additionally, since isotactic PS can form a non-covalent crosslinked matrix with CNTs, the *T_g_* of CNTs/isotactic PS composite materials increases with the addition of CNTs. This study illustrates that the dispersion of CNTs in a polymer matrix can be controlled by adjusting the conformational regularity of the polymer itself.

Improving the dispersion performance of CNTs in pure PS through simple adsorption is limited. Therefore, it can be attempted to chemically modify PS before using it for the non-covalent modification of CNTs. Yan et al. [4] used pyrene-terminated PS (PyPS) prepared using an anionic polymerization method as a dispersant for SWCNTs. They investigated the dispersion performance of PyPS-modified CNTs and the interactions between PyPS and CNTs. The results showed that the introduction of PyPS made the dispersion of CNTs in chloroform very stable and uniform, with PyPS and CNTs interacting through π–π stacking and adsorbing on the surface of CNTs. The current percolation threshold of PS composites prepared using PyPS as a dispersant for SWCNTs decreased from 0.28 wt.% without PyPS to 0.095 wt.%. It is known that non-conjugated polymer PS has non-covalent interactions with CNTs, but the interaction between conjugated polymers and CNTs is stronger [116,117]. Zou et al. [118] suggested that copolymers consisting of non-conjugated polymer segments and conjugated polymer segments should be excellent dispersants and stabilizers for CNTs. Therefore, they synthesized a copolymer P3HT-b-PS composed of polythiophene (P3HT) and PS. The study found that the P3HT in P3HT-b-PS could form a close interaction with CNTs, and PS could protect and provide good solubility for CNTs. This method of CNT modification does not involve chemical bonding, so it does not disrupt the π–electron structure of CNTs and does not affect the inherent properties of CNTs. Furthermore, the research demonstrated that the solubility of CNTs can be customized by introducing different compatibilizing polymers into block copolymers. Due to the strong interaction between CNTs and the conjugated block in the copolymer, the dispersed CNT solution can be dried and easily redispersed in a solution. They provided a universal method for dispersing CNTs.

### 3.3. Other Methods

Methods for surface modification of CNTs in PS mainly involve covalent and non-covalent modifications of PS and related polymers, along with some other surface modification methods for CNTs. Carboxylation of CNTs’ surfaces can be achieved through simple ultraviolet ozone (UVO) irradiation [119,120], enhancing the dispersion of CNTs. Ayesh et al. [121] demonstrated that UVO irradiation for a certain duration could functionalize the surface of CNTs. This not only improved the dispersion of CNTs in organic solvents but also enhanced the rheological and optoelectronic properties of PS composite materials. Choosing polymers with good affinity to PS is also an ideal modification material, and polyaniline is one such polymer. Sarvi et al. [122] performed non-covalent in situ polymerization modification of aniline monomers in the presence of MWCNTs. Due to the good affinity between polyaniline and PS, the prepared polyaniline-modified CNTs exhibited better dispersion when introduced into the PS matrix compared to unmodified ones, with a lower percolation threshold for the prepared composite material.

Covalently grafting some non-PS-related long chains on the surface of CNTs is also advantageous for their dispersion. Wang et al. [123] used oleic acid for surface modification of CNTs. Firstly, CNTs were acid-oxidized, then reacted with formaldehyde in an alkaline environment to generate hydroxymethyl, and finally reacted with oleic acid in xylene to obtain oleic acid covalently modified MWCNTs. The introduction of oleic acid did not disrupt the original structure of CNTs and provided them with good stability and dispersion. Additionally, oleic acid modification improved the interface interaction between the PS matrix and MWCNTs. Amr et al. [124] used 1-octadecanol for surface covalent modification of carboxylated CNTs, which were then introduced into PS. When the load of 1-octadecanol-modified CNTs in PS was low (0.5 wt.%), they exhibited good dispersion in PS. Non-covalent modification using surface-active agents unrelated to PS or covalent bonding modification with surface-active agents without benzene rings can also interact with CNTs. Sun et al. [125] prepared the twin surfactant 6,60-(butane-1,4-diylbis(oxy))bis(3-nonylbenzenesulfonic acid) (9BA-4-9BA) for CNTs’ dispersion. The study showed that the benzene rings in 9BA-4-9BA could adsorb on the surface of CNTs through π–electron interactions, dispersing CNTs uniformly and stably in toluene. The prepared modified CNTs/PS composite material exhibited better thermal stability and conductivity than pure PS. Mallakpour et al. [126] used leucine to form amide chemical bonds with carboxylated CNTs, reducing the aggregation of CNTs in PS. The modified CNTs exhibited amide–π and π–π interactions with PS, enhancing the compatibility between CNTs and PS. Moreover, the introduction of CNTs improved the conductivity and thermal stability of PS. Section 2.2 introduced the method of modifying CNTs by adsorbing PS particles on their surfaces. In fact, the adsorption of CNTs on the surface of PS microspheres is also beneficial for the dispersion of CNTs in the PS matrix and the preparation of functional composite materials. Li et al. [127] first prepared PS microspheres using the stabilizer poly(vinyl pyrrolidone) and the initiator AIBN. Then, they surface-hydrophilized MWCNTs by acid treatment, allowing them to disperse uniformly in water. Finally, MWCNTs with good dispersibility in water could spontaneously adsorb and self-assemble onto the surface of PS microspheres because this self-assembly behavior is energetically favorable in colloid thermodynamics. The MWCNTs adsorbed on the surface of PS microspheres acted as steric stabilizers, promoting the stability of microspheres, ultimately forming PS/MWCNTs composite microspheres. This method is simple, and the loading amount of MWCNTs on the surface of PS microspheres is controllable.

Melt blending is a commonly used method for industrial production of thermoplastic composites, so developing methods to enhance the dispersion of CNTs based on melt blending processes has potential industrial application value. The addition of some carbon nanomaterial surface modifiers during the melt blending process is an effective approach. Bellayer et al. [128] used melt extrusion to prepare MWCNTs/PS (polystyrene) composite materials containing a compatibilizer (tri-alkyl imidazolium tetrafluoroborate). MWCNTs could interact with the compatibilizer, forming π–cations and nanotube–imidazole interactions, effectively preventing the agglomeration of MWCNTs and promoting their dispersion in PS. Ionic liquids have good thermal stability, solubility, and ionic conductivity, composed of anion–cation pairs that can interact with CNTs. Soares et al. [129] prepared PS/ethylene–vinyl acetate (EVA) composite materials modified with ionic-liquid-modified CNTs using a melt method, and the study found that compared to CNTs/PS/EVA composite materials without ionic modification, the modified composite exhibited higher conductivity and a lower percolation threshold. This is mainly due to the non-covalent modification of the ionic liquid, which helps disperse CNTs in the polymer matrix and facilitates the formation of conductive pathways. Furthermore, the prepared ionic-liquid-modified CNTs polymer composite material also had better electromagnetic interference shielding capabilities. The viscosity of the polymer during the melt blending process can influence the dispersion of CNTs [73]. Zhang et al. [130] used melt blending to prepare conductive polymer composites (CPCs) of CNTs/polyphenylene ether (PPO)/PS and studied the influence of the viscosity of the PPO and PS matrix after blending on the dispersion of CNTs. The results showed that the dispersion of CNTs was optimal when the viscosity of the PPO and PS matrix was in the medium range, with very low percolation thresholds and resistivity lower by nine orders of magnitude than PS/2% CNTs. During the melt blending process, thermal and shear actions caused PS to wrap around the surface of CNTs through π–π stacking. Zhou et al. [131] used melt blending to investigate the coverage of PS on the surface of CNTs at different melt temperatures and times, determined the optimal processing parameters, and found that PS-wrapped CNTs could be well dispersed in tetrahydrofuran solution. In addition, mechanical testing revealed that, compared to pure PS and PS composites with original CNTs, melt-blending-modified CNTs/PS composites exhibited significantly enhanced mechanical properties. Apart from the mentioned methods, effective processing techniques, such as simple mechanical rolling mixing of CNTs and PS powders, can also promote the dispersion of CNTs in the PS matrix. Sachdev et al. [132] dispersed CNTs in PS using the mechanical rolling mixing method. Initially, they opened the agglomerates of CNTs at low speed with a drum mixer, followed by uniform dispersion of CNTs in the matrix of PS powder at high speed. Finally, they directly prepared CNTs/PS composite materials using a hot press. The prepared composite materials showed lower percolation thresholds and excellent electromagnetic shielding effects.

### 3.4. Comparisons of the Functionalization Methods

Table 2 provides a comparison and summary of the three categories of CNT functionalization for CNT/PS composites, including their characterizations, mechanisms, and advantages and disadvantages. Surface modification of CNTs in polymers is not limited to the mentioned methods, but Table 2 almost covers the surface modification methods of CNTs in PS. Covalent grafting of CNTs using PS-related polymers involves chemical reactions between CNTs and the grafting polymer, introducing strong interactions but partially destroying the atomic structure of CNTs. In contrast, non-covalent functionalization maintains the strong carbon structure, while the interaction between CNTs and functionalization reagents is relatively weak. Table 2 can serve as a guide for surface modification methods of CNTs in polymers, especially in PS and other aromatic polymers. Table 3 supplements Table 2 and summarizes the impact of different CNTs’ functionalization methods on their dispersion and polymer composite properties.

## 4. The Properties of CNTs/PS Composite Materials

### 4.1. Mechanical Properties

**Figure 5:**
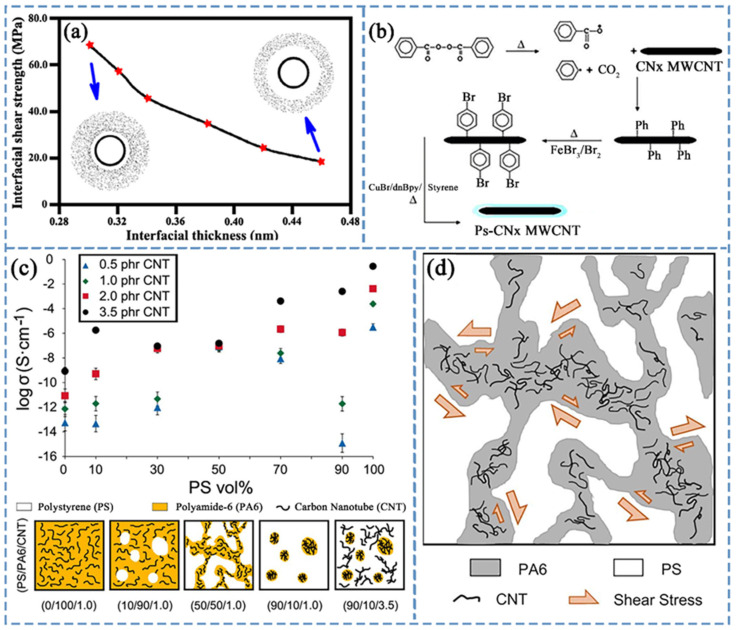
(**a**) Relationship between interfacial shear strength and interfacial layer thickness. Reproduced with permission [136]. Copyright 2012, Elsevier, Ltd.; (**b**) Synthesis diagram of Ps-CNx MWCNTs. Reproduced with permission [143]. Copyright 2006, Elsevier B.V.; (**c**) (**top**) DC electrical conductivity of PS/PA6/CNT composites with different CNT contents and (**bottom**) schematic representation of morphological evolution of PS/PA6/CNT composites. Reproduced with permission [147]. Copyright 2017, Elsevier, Ltd.; (**d**) Shear forces experienced by PS/PA6/CNT composites during the melt mixing process (shape size represents the magnitude of the value). Reproduced with permission [147]. Copyright 2017, Elsevier, Ltd.

CNTs possess excellent tensile strength and elastic modulus, making them a promising polymer material reinforcement additive [133,134]. The mechanical properties of CNT–polymer composites are dominated by interface characteristics. Moreover, compared to non-covalent bonding, covalent bonding is more favorable for enhancing the mechanical performance of polymer composites. This is mainly due to the fact that covalent bonding at the interface not only strengthens the interaction between CNTs and the polymer matrix but also facilitates load transfer across the interface [135]. Wernik et al. [136], through the establishment of a pullout model, explored the relationship between interfacial shear strength and interfacial thickness. When the interface involves van der Waals interactions, the shear strength of the interface decreases with an increase in the thickness of the interface layer (as shown in Figure 5a). Xiong et al. [137] reached conclusions similar to those of Wernik et al. Through simulations of the mechanical properties of CNT composites, Xiong and colleagues concluded that the interfacial shear strength is determined by the intrinsic properties of the polymer and CNTs, and the efficiency of load transfer at the interface is highly correlated with the interface strength. Additionally, they found a dependence relationship between interfacial shear strength and interface thickness, stating that a smaller interface thickness leads to stronger van der Waals interactions and pullout forces, resulting in a larger interfacial shear strength. Rafiee et al. [138] simulated interfaces with van der Waals force interactions and found that the efficiency of load transfer at the interface and the macroscopic properties of the composite material are governed by the intrinsic characteristics of CNTs. Therefore, controlling the thickness of the interface and the strength of binding/interactions is crucial for ensuring the efficiency of load transfer at the interface and the macroscopic mechanical properties of polymer nanocomposites.

Localized strain and fracture in polymer composites often occur at the weakest points in the matrix, necessitating a microscopic analysis to understand the fracture failure mechanism of polymer composites. During the tensile process, for brittle PS, localized stress concentration leads to the initiation and propagation of cracks, ultimately resulting in rapid fracture [139]. Lin et al. [97], in their study, regarded cracks as a mechanism for dissipating mechanical energy applied externally to the matrix and investigated the impact of introducing functionalized CNTs into PS on its mechanical properties. For unmodified CNTs introduced into PS, cracks generated during the tensile process continued to grow in length and width, ultimately leading to fracture. However, for PS with covalently grafted CNTs introduced after modification, cracks appearing under lower stress concentration conditions remained short and narrow, less prone to growth and fracture. This mechanism, by resisting external forces through this dissipative process, enhanced the mechanical properties of PS. The realization of the above energy dissipation mechanism is closely related to the good compatibility of functionalized CNTs with PS and the excellent dispersion of CNTs in PS. Nayak et al. [140], through a solvent-free functionalization method, covalently grafted 4-ethylaniline onto the sidewalls of CNTs, then introduced it into PS, and prepared CNTs/PS composite materials using a solution casting method. The study demonstrated that the functionalization of CNTs improved their dispersion and compatibility in PS; thus, the CNTs/PS composite material after functionalization exhibited better mechanical (tensile strength and flexural modulus) and electrical properties. Additionally, Yuan et al. [141] used the in situ polymerization of PS to modify MWCNTs through encapsulation and then prepared CNTs/PS composite materials. The research found that the PS wrapped on the surface of MWCNTs improved their compatibility with PS and enhanced the interface interaction, resulting in significantly improved mechanical properties compared to pure PS and the original CNTs/PS composite material. Furthermore, the dynamic mechanical properties of CNTs/PS composite materials are closely related to the dispersion of CNTs and their interaction with PS. Putz et al. [142] investigated the dynamic mechanical properties of functionalized CNTs/PS composite materials with temperature variations. The study revealed that when the CNT content was less than 0.1 wt.%, the composite modulus of the CNTs/PS composite material exceeded the predicted value of the Halpin–Tsai model, and the loss tangent of the composite material was the same as that of pure PS, indicating similar elasticity. However, at higher concentrations, the nanocomposite material was more elastic than pure PS, but the composite modulus was lower than the predicted value of the Halpin–Tsai model. The research results indicate that the dynamic mechanical properties of functionalized CNTs/PS composite materials are highly sensitive to the dispersion of CNTs in PS, aggregate size, and the interface interaction between CNTs and PS.

In situ covalent grafting modification of CNTs is effective in enhancing their mechanical properties. Fragneaud et al. [143] employed the ATRP method to covalently graft PS onto the surface of nitrogen-doped MWCNTs (CNx MWCNTs). The grafting process mainly involves the following steps: preparing nitrogen-doped MWCNTs through chemical vapor deposition, grafting aromatic rings during the ATRP reaction, further bromination, and finally, using bromine molecules as initiators for the ATRP reaction of styrene on the surface of CNx MWCNTs (Figure 5b illustrates the schematic synthesis of PS-CNx MWCNTs). The study demonstrated that the prepared CNx MWCNTs have a uniform amorphous PS layer covering the surface with a thickness of 3–5 nm. In situ covalently modified nitrogen-doped MWCNTs can be used to reinforce polymer manufacturing and exhibit excellent mechanical properties. Subsequent research by Fragneaud et al. [144] showed that grafting short-chain polymers onto CNTs can improve the dispersion of CNTs in the polymer matrix, increase the composite material’s Young’s modulus (at temperatures below the *T_g_* of the polymer), and improve the stress fracture of the composite material. It was also found that grafting low-molecular-weight polymers can lead to the plasticization of the CNTs’ surface matrix. The mechanical properties of PS composite materials are influenced by both the molecular weight and grafting density of PS grafted onto the CNTs surface. Chadwick et al. [94] synthesized PS-modified SWCNTs (PS-SWCNTs) using covalent bonding methods and discussed the impact of PS molecular weight and grafting density on the macroscopic mechanical properties of PS-SWCNTs. Research data indicated that when the molecular weight of PS is low, the mechanical properties of PS-SWCNTs are better, and at lower molecular weights, grafting density is the main influencing factor. The shear strength between CNTs and stress transfer between them are controlled by the grafting density and are independent of the molecular weight of PS molecular chains. Therefore, the mechanical properties of PS-SWCNTs are primarily influenced by the grafting density. As the molecular weight decreases, the grafting density increases, but the molecular weight cannot fall below a threshold because the interface strength requires van der Waals forces and stress transfer between CNTs, which require PS molecular chains of a certain molecular weight to provide.

Although in-situ covalent grafting modification benefits the improvement of the mechanical properties of CNTs/PS composite materials, it tends to lower their *T_g_*. Research by relevant scholars indicates that the *T_g_* of CNTs/PS composite materials is related to the molecular weight of PS, and the presence of CNTs is unfavorable for the formation of larger molecular weight PS from styrene monomers. Amr et al. [124] prepared CNTs/PS composite materials covalently modified with 1-octadecanol. When the modified CNTs dosage was 0.5 wt.%, Young’s modulus increased by about 20% compared to the original CNTs/PS composite material. However, due to the polymerization of styrene monomers in the presence of CNTs, the molecular weight of PS decreased, leading to a decrease in the *T_g_*. Additionally, melt rheology studies found that original CNTs increased the viscoelasticity of PS, while modified CNTs acted as plasticizers. Acid-oxidized modified CNTs had many similar effects to those of 1-octadecanol covalently modified CNTs [145]. Among various surface modifications of CNTs, carboxylation modification is one of the most commonly used methods. However, experimental results indicate that after covalent grafting modification of CNTs with PS-related polymers, the prepared polymer composite materials exhibit a higher *T_g_*, better thermal stability, as well as superior tensile and impact properties [105]. When materials are subjected to cyclic loading, their failure stress is often lower than the stress failure level under static conditions, which is related to the creep properties of the material. The introduction of CNTs into PS affects its creep behavior, and the creep behavior of the material can be used to evaluate its mechanical and deformation properties. Jia et al. [146] studied the recovery and short-term creep behavior of MWCNTs/PS composite materials under cyclic tensile conditions. The results showed that for both modified and unmodified CNTs/PS composite materials, the creep strain decreased with an increase in CNTs content, a decrease in stress and temperature, and the reduction in creep strain became more significant with an increasing number of cycles. The above trends were more pronounced at high temperatures and high stresses. The mechanism by which the introduction of CNTs affects the creep performance of PS is that CNTs form a network structure with PS molecular chains, restricting the movement of molecular chains, reducing creep strain, and increasing the recovery rate.

### 4.2. Electrical Conductivity

PCs are an important class of functional composite materials achieved primarily by incorporating conductive fillers into polymers [148,149,150]. Interactions at the interface of polymer nanocomposites can influence their electrical conductivity [151]. This is manifested in both beneficial and detrimental effects on the electrical performance of nanocomposite materials [152]. Hoseini et al. [147] introduced CNTs into a mixture of PS and polyamide-6 (PA6) through melt blending, studying the morphology, filler content, and interactions between filler and polymer to investigate percolation behavior and electrical conductivity of the blended composite material. The results revealed that, at the same CNTs content, the electrical conductivity of the co-continuous phase composite material formed by PS/PA6 (50/50) and CNTs was about four orders of magnitude lower than that of the non-co-continuous phase composite material formed by PS/PA6 (90/10) (see Figure 5c (top)). This was mainly due to the better thermodynamic affinity between PA and CNTs, leading to a preference for CNTs to associate with PA6. In PS/PA6 (50/50), although a continuous phase of PA6 and CNTs was formed, the dispersion of CNTs in this continuous phase was poor because CNTs in the PA6 phase experienced less shear force during the melt blending process (see Figure 5d). However, in PS/PA6 (90/10), primary and secondary percolation mechanisms were established, where CNTs in the PS phase served as bridges for CNTs in the PA6 phase, promoting an enhancement in electrical conductivity. Figure 5c (bottom) also illustrates the phase morphology and structure in composite materials with different CNTs contents and various PS-to-PA ratios.

When the content of CNTs introduced into the polymer is relatively low, an increase in the interface thickness in polymer nanocomposites will lower their percolation threshold. However, when the content of CNTs exceeds the percolation threshold, the effective electrical conductivity of the nanocomposite material is significantly influenced by the conductivity at the interface [153]. The electrical conductivity of CNTs–polymer composite materials is closely related to the distribution of CNTs within the polymer matrix. When CNTs are more aligned, they have a higher percolation threshold compared to randomly oriented CNTs, mainly because there is less interface contact between CNTs, resulting in poorer macroscopic electrical conductivity of the polymer nanocomposite material [154]. Furthermore, the aggregation of CNTs in polymer nanocomposites can affect their electrical conductivity, and the crystallization and wrapping of polymer chains at the interface with CNTs may also have adverse effects on electrical performance [152].

The electrical conductivity of CNTs composite materials is closely related to the dispersion of CNTs in the polymer matrix and their compatibility with the matrix. Good dispersion and compatibility of CNTs can significantly reduce the required content of CNTs to reach the percolation threshold for polymer conductivity [155]. Zhang et al. [141] studied the electrical conductivity of blends of different mass ratios of PPO and PS filled with CNTs. By changing the mass ratio of PPO and PS, the viscosity of the blend can be controlled, and the viscosity of the blend affects the dispersion of CNTs in the blend matrix. The study found that when the PPO and PS blend had medium viscosity, the optimal dispersion state of CNTs could be achieved. The percolation threshold of PPO/PS (35/65)/CNTs composite material was 63% lower than that of PS/CNTs, and the resistivity of PPO/PS (35/65)/2% CNTs composite material was nine orders of magnitude lower than that of PS/2% CNTs. Additionally, Wang et al. [156] successfully prepared PS composite materials filled with different contents of SWCNTs using an improved suspension polymerization technique. SWCNTs and styrene monomers underwent an in -situ chemical bonding reaction under the conditions of the presence of free radicals, resulting in SWCNTs adsorbed and encapsulated by PS (SWCNTs–PS). SWCNTs–PS could dissolve in toluene and form a stable solution that could be maintained for over a month. Furthermore, when the load of CNTs was 1%, its volume resistivity decreased by 10 orders of magnitude compared to pure PS. Moreover, Chang et al. [157], by subjecting SWCNTs to heat treatment to remove amorphous carbon and residual catalysts, introduced them into PS. They obtained SWCNTs/PS composite materials with different mass fractions through solution mixing, drying, and hot pressing. The study found that the CNTs/PS composite material after heat treatment had a lower percolation threshold (less than 0.3 wt.%) and higher conductivity than the original CNTs/PS composite material, indicating better dispersion of CNTs in PS after annealing. Raman spectroscopy under tension showed good stress transfer from the polymer matrix to CNTs. In addition, the modulus of the annealed CNTs/PS composite material slightly increased, but the specific reasons remain unexplained.

The preparation method of CNTs/PS composite materials can affect the dispersion of CNTs in the matrix, thereby influencing their electrical conductivity. Zhang et al. [158] employed two methods to prepare MWCNTs/PS conductive polymer composites. The first method involved in situ polymerization in the presence of MWCNTs, while the second method utilized a solution blending approach. A comparative study on the percolation behavior and corresponding resistance characteristics to organic vapors was conducted for the composites prepared by both methods. The results revealed that the in situ polymerization method was more favorable for the dispersion of CNTs in PS. Furthermore, the conductive composites prepared within a filler mass fraction range of 5–15 wt.% exhibited higher response speed and sensitivity. Additionally, an increase in temperature facilitated an improvement in the response rate but led to a decrease in the maximum response. Faraguna et al. [159] found that solution mixing resulted in less polymer matrix degradation and maintained the length of MWCNTs, while melt mixing caused a reduction in MWCNT length, resulting in a relatively higher current percolation threshold. The *T_g_* of MWCNTs/PS composites prepared by melt blending was almost unaffected by MWCNTs modification. However, *T_g_* was influenced by PS molecular weight, with a degradation in molecular weight observed during melt blending, leading to a reduction in the composite’s *T_g_*. A transition to lower temperatures was observed for composites with lower-molecular-weight PS. In summary, MWCNTs/PS composites prepared using the masterbatch and solution methods exhibited optimal performance, including excellent dispersion, high conductivity, low current percolation threshold, high rheological response, low rheological percolation threshold, and higher storage modulus. Moreover, Zhou et al. [160] employed masterbatch dilution and microneedle injection molding techniques to prepare CNTs/PS composites and investigated the influence of process parameters (melt temperature and injection speed, etc.) on the electrical conductivity and microstructure of the composites. The study found that melt temperature significantly affected the composite’s electrical conductivity, with conductivity being positively correlated with the amount of added CNTs. Additionally, experimental and simulation results indicated that CNTs preferred to orient along the flow direction of the molten composite, mainly due to stretching effects and higher shear forces, and this orientation became more pronounced with a decrease in mold thickness.

SDS can enhance the dispersibility of CNTs, thereby improving the electrical conductivity of CNTs/PS composite materials. Wu et al. [161] employed the anionic surfactant SDS in an emulsion polymerization method to prepare monodisperse PS microspheres of approximately 230 nm. CNTs were introduced into the suspension of PS microspheres, and MWCNTs/PS nanocomposite materials were prepared using a latex method. The MWCNTs exhibited good dispersion in PS, and with increasing CNT content, the *T_g_* of the composite material gradually increased, mainly due to the interactions between CNTs and PS restricting the movement of PS polymer chains. Additionally, when the CNT content in the composite material was 1.5 wt.% and 6.5 wt.%, the electrical conductivity was higher by 4 and 10 orders of magnitude, respectively, compared to pure PS. Unfortunately, the introduction of SDS has a negative impact on the conductivity of CNTs. Yu et al. [162] investigated the influence of SDS on the dispersion of MWCNTs and the performance of the resulting composite material. The study found that while SDS could debundle CNTs and prevent their re-aggregation, the presence of SDS affected the charge transfer at the junctions of CNTs. Therefore, it is essential to find a balance between CNT dispersion and conductivity with the appropriate amount of SDS. Through the latex method for preparing CNTs composites, the research discovered that when the mass ratio of CNTs to SDS was approximately 1:1.5, the decrease in the electrical conductivity of the composite material was minimal, and the dispersion of CNTs in SDS reached its maximum. Moreover, the volume conductivity of the SDS-modified MWCNTs/PS composite material reached 1 S/m when the CNTs content reached the current percolation threshold of 1.5 wt.%, representing an increase of 10 orders of magnitude compared to pure PS.

**Figure 6:**
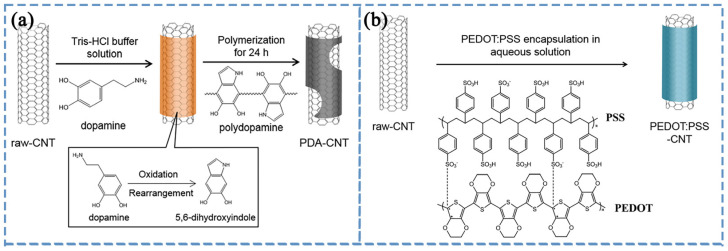
(**a**) Schematic diagram of PDA and (**b**) PEDOT:PSS-modified CNTs. Reproduced with permission [163]. Copyright 2021, MDPI.

Introducing conductive polymers into CNTs/PS enhances their electrical conductivity. Song et al. [163] first prepared two types of non-covalently surface-functionalized CNTs, namely polydopamine (PDA)-modified CNTs (as shown in Figure 6a) and poly(3,4-ethylenedioxythiophene): poly(styrene sulfonate) (PEDOT:PSS)-modified CNTs (as shown in Figure 6b), and then successfully prepared PS composite materials with uniformly distributed CNTs through emulsion methods. The electrical conductivity of these materials was studied, and the results showed that introducing PEDOT:PSS-modified CNTs into PS improved the electrical conductivity. The main reason is that the inherent conductivity of PEDOT:PSS facilitates the formation of a conductive network within the PS matrix. Additionally, the study found that introducing ethylene glycol during the modification of PEDOT:PSS-modified CNTs further enhances the electrical conductivity of PS. In addition to introducing conductive polymers, inorganic conductive nanofillers can also be introduced. Graphene nanosheets (GNSs) are outstanding conductive nanofillers. Pang et al. [164] prepared GNSs/PS and CNTs/PS composite materials using a combination of solution mixing and hot pressing. The study showed that the time required to reach the percolation threshold for the prepared composite materials decreased with increasing filler loading, annealing temperature, and electric field strength. Moreover, compared to CNTs, GNSs more easily formed conductive paths in PS composite materials. Furthermore, under the action of an electric field, both CNTs and GNSs can overcome the hindrance of the polymer to form a conductive network, and the ease of forming the conductive network is closely related to the geometric shape of the conductive particles. Therefore, GNSs can be used in conjunction with CNTs to synergistically enhance the electrical conductivity of PS composite materials. Patole et al. [165] modified a mixture of graphene and CNTs in a water solution using the hydrophobic agent 1-pentanol and the surfactant SDS. They then added styrene monomers and initiators and prepared CNTs/graphene/PS composite materials through emulsion polymerization in the solution. As CNTs can act as bridges between graphene sheets, the synergistic effect of the two fillers imparts excellent electrical, mechanical, and thermal properties to the PS composite material.

CNTs can also be used in the manufacturing of electrochemical luminescence sensors. Li et al. [166] successfully incorporated CNTs into partially sulfonated polystyrene (PSP) to create an electrochemical luminescence sensor based on PSP/CNTs composite films. The study found that CNTs played a role in providing a conductive pathway, accelerating electron transfer, and serving as a suitable matrix. Moreover, due to the electrocatalytic activity of CNTs, the electrochemical luminescence intensity of the prepared composite film was more than three times that of pure PSP. Additionally, in the study by Shah et al. [167], it was observed that the dielectric loss and dielectric constant of CNTs/PS composite materials increased with the loading of MWCNTs in PS. The alternating current conductivity increased with temperature, attributed to the π–electron transitions in MWCNTs.

### 4.3. Electromagnetic Shielding Performance

Conductive polymer-based composites possess advantages required for electromagnetic interference shielding materials, such as lightweight, corrosion resistance, durability, and excellent mechanical properties [168]. They have found widespread applications in electromagnetic interference (EMI) shielding, with CNTs being favored in the preparation of electrically conductive polymer nanocomposites (CPNs) [132,169,170]. Constructing segregated structures in CPNs is an effective method for preparing electrically conductive polymer composites with excellent EMI shielding performance [171,172]. The presence of segregated structures eliminates the need for extensive consideration of CNTs dispersion during the preparation process, making the manufacturing method more straightforward. Keshmiri et al. [173] proposed a method for preparing high-performance electromagnetic shielding conductive polymers. They first prepared PS microspheres using the anionic surfactant SDS. Subsequently, they dispersed the PS microspheres, PEDOT:PSS, and CNTs in a water solution. The resulting mixture was then successfully processed through filtration, methanol treatment, and hot pressing to create a segregated structure in the CNTs/PS conductive nanocomposite material. The percolation threshold of the prepared conductive nanocomposite material was found to be 0.009 vol%, and the introduction of PEDOT:PSS further improved the conductive network by enhancing contact and dispersion between CNTs (π–π interactions between PEDOT:PSS and CNTs facilitated a more complete encapsulation of CNTs on the surface of PS microspheres). The electrical conductivity and EMI shielding effectiveness (SE) of the PS/CNTs/PEDOT:PSS (100:2:4 *w*/*w*/*w*) were measured at 2.352 S/cm and 55.7 dB/mm, respectively, with absorption being the dominant mechanism for EMI shielding. Xie et al. [174], based on CNTs and expandable PS beads, successfully prepared a CNTs/PS composite foam with a porous segregated structure using microwave-assisted foaming. The percolation threshold of the prepared porous segregated structure composite foam was as low as 0.0014 vol%. With a CNTs loading of 0.046 vol%, the EMI SE dominated by absorption reached 211.5 dB cm^3^/g at 12.4 GHz. Additionally, the sintering effect of microwaves softened and melted the PS beads, promoting mutual penetration and significantly enhancing the mechanical properties of the composite foam, which increased with the CNTs loading.

**Figure 7:**
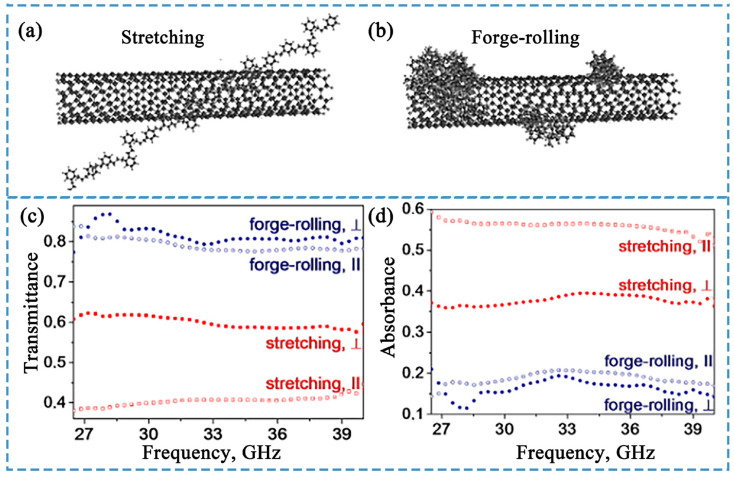
State of CNTs on the surface of PS in CNTs/PS composite materials prepared by (**a**) stretching method and (**b**) forge-rolling method, and their impact on microwave (**c**) transmission and (**d**) absorption. Reproduced with permission [179]. Copyright 2016, Elsevier Ltd.

CNTs exhibit magnetic anisotropy as a material [175]. PS is a magnetically isotropic material, but the susceptibility of PS to magnetization is greatly influenced by the orientation of the benzene rings. Additionally, research indicates a strong interface interaction between CNTs and aromatic polymers. This interaction is influenced by the arrangement of CNTs and polymer molecular chains. Experimental and simulation results demonstrate that the interface interaction is strongest when the stretching direction of PS aligns with the direction of CNTs [176,177,178]. Based on these findings, Makarova et al. [179] prepared CNTs/PS composite materials with the same CNTs content using both forge-rolling and stretching methods, studying their magnetic properties. The results showed that Raman spectroscopy revealed π–π interactions between CNTs and PS molecules, and the interface interaction and arrangement of CNTs in PS were closely related to the preparation method of CNTs/PS composite materials. As shown in Figure 7a,b, the stretching process allowed the PS molecular chains to make better contact with CNTs. The benzene rings in the PS molecular chains were approximately parallel to the CNTs, enhancing intermolecular forces. Moreover, PS could better encapsulate the surface of CNTs. In comparison to PS/CNTs composite materials prepared by forge-rolling, where PS on the surface of CNTs is disordered, the orientation of the benzene rings is also disordered, and the interaction force between them is relatively weaker. Electromagnetic property testing demonstrated that PS/CNTs composite materials prepared by forge-rolling had no significant impact on the transmission and absorption of microwaves regardless of the polarization direction of the electric field (the polarization direction of the electric field is perpendicular to the direction of electromagnetic wave propagation). In contrast, PS/CNTs composite materials prepared by stretching were closely related to the polarization direction of the electric field, as shown in Figure 7c,d. Therefore, the above research demonstrates that magnetic measurements of CNTs/PS composite materials can provide information about the orientation of CNTs and the surrounding PS. This non-destructive measurement method can be applied to the study of CNTs/PS composite materials and can be extended to some nanocomposites with strong correlations. Similar research has also been reported in another work by Makarova [180].

The orientation of CNTs in PS is influenced by the injection molding process, which can result in changes in the EMI shielding performance of CNTs/PS composite materials. Mahmoodi et al. [181] found that the injection molding process conditions during the preparation of CNTs/PS composite materials affect the electrical conductivity and EMI SE of the composite material. Adjusting injection molding process conditions such as injection speed, pressure, and temperature can cause a seven-order-of-magnitude change in the volume resistivity of the composite material. Moreover, the injection molding process can influence the orientation of CNTs in PS. The study revealed that the EMI SE of the composite material decreases with an increase in the degree of CNT orientation. Arjmand et al. [182] conducted a comparative study on the electrical conductivity, EMI shielding, and dielectric properties of MWCNTs/PS composite materials prepared by compression molding and injection molding, yielding conclusions similar to those of Mahmoodi et al. [181]. Arjmand et al.’s findings indicate that, compared to injection molding, composite materials prepared by compression molding exhibit better electromagnetic interference shielding effectiveness, lower percolation threshold, higher electrical conductivity, and higher dielectric constant. The main reason for these results is that injection molding aligns CNTs, reducing the probability of contact between CNTs. In contrast, CNTs in the PS matrix exhibit a random distribution during compression molding. Additionally, the comparison of EMI shielding performance in the above results demonstrates a significant enhancement in the composite material’s EMI SE with filler connectivity. However, even in the absence of filler connectivity, the composite material can still exhibit EMI shielding performance.

Producing EMI shielding conductive polymer composites based on CNTs and PS is straightforward. Qavamnia et al. [183] used the cost-effective, efficient, and simple electrospinning technique to prepare CNTs/PS composite material fibers. The study investigated the influence of CNTs loading on its electromagnetic shielding, conductivity, and thermal stability. As the CNTs content increased, the electromagnetic shielding and conductivity of the composite material gradually improved due to the formation of a conductive network within the matrix. Additionally, the introduction of CNTs increased the thermal stability of PS. Yang et al. [184] utilized a microsprayer to spray a mixture of toluene containing ultrasonically dispersed CNTs and a foaming agent onto a flat surface. After thermal curing, foaming agent release was induced through heat pressing, ultimately forming CNTs–PS foam composite material. The prepared CNTs–PS polymer foam composite material can be used for EMI shielding, achieving an EMI SE of approximately 20 dB when the CNTs loading is 7 wt.%. The primary EMI shielding mechanism is the reflection of electromagnetic radiation. Table 4 is a comparison and summary of the electromagnetic shielding properties of CNTs/polystyrene composites prepared by the above different methods.

### 4.4. Thermal Conductivity and Thermal Stability

The low thermal conductivity of PS limits its applications in the field of heat transfer composite materials [185]. Therefore, enhancing the thermal conductivity of PS is of significant importance for expanding its applications in heat transfer. The introduction of CNTs alone in PS can have a positive impact on its thermal stability. Sen et al. [176] prepared carboxyl-functionalized modified MWCNTs/PS nanocomposite materials through a combination of ultrasound and solvent mixing. The study demonstrated that the introduction of carboxyl-functionalized modified MWCNTs can significantly enhance the thermal stability of PS. CNTs can also synergistically improve the thermal stability of PS with other fillers, even imparting additional beneficial properties to PS. Hatui et al. [186] reported the synergistic enhancement effect of expanded graphite (EG) and MWCNTs on the properties of PS. Compared to using each material separately, the synergistic enhancement effect of both materials on PS is more significant, leading to a better performance in terms of *T_g_*, storage modulus, and thermal stability. Furthermore, EG and MWCNTs can promote their respective dispersion in PS. Chaudhary et al. [187] prepared CNTs/starch/PS composite materials through a solution casting method. Experimental studies showed that the prepared composite materials have higher thermal stability and electrical conductivity compared to pure PS. Additionally, the biodegradation rate of the prepared composite material is higher, contributing to the recyclability of PS composite materials. The introduction of CNTs can significantly enhance the thermal conductivity of PS, but in some areas, polymer materials need to have lower thermal conductivity, such as devices used in factories with heating or cooling, insulation, and the exterior walls of buildings. Gong et al. [188] used supercritical carbon dioxide foaming technology to prepare CNTs/PS foam composite materials, which demonstrated outstanding thermal insulation effects even in the absence of insulating gases.

The primary mode of heat transfer is lattice vibration, and due to poor coupling of vibrations, high thermal resistance often forms at interfaces [189]. Huxtable et al.’s research indicates that the thermal conductivity at interfaces is extremely low, and its thermal resistance is equivalent to that of a 20 nm thick polymer [190]. Gojny et al. [191] believe that introducing MWCNTs into nanocomposites has great potential in improving their thermal performance. This is because the interfaces in MWCNTs composite materials not only have low scattering potential for phonons, but the shielding effect of the inner layers of MWCNTs is also advantageous for phonon propagation and reduces phonon coupling losses at the interface. Relevant studies show that introducing covalent bonds at the interface can reduce phonon scattering at the interface, thereby reducing the thermal resistance at the interface [192]. However, covalent modification of CNTs can lower their intrinsic thermal properties, so the degree of covalent modification of CNTs should not be too high [193]. Research indicates that interface thermal resistance increases as the temperature decreases. Jakubinek et al. [194] revealed the relationship between the thermal conductivity of SWCNT/PS composite materials and temperature (studied in the temperature range of 140–360 K). The study found that the thermal conductivity of SWCNT/PS increases with increasing temperature. This is mainly because, at lower temperatures, the interface thermal resistance increases, and the enhancement factor decreases. However, when the temperature exceeds 300 K, the enhancement factor becomes relatively stable. Therefore, it confirms that temperature affects the thermal conductivity of SWCNT/PS composite materials by influencing interface thermal resistance.

The preparation method of CNTs/PS composite materials can impact their thermal stability, and emulsion polymerization is a method for producing CNTs/PS composite materials with good thermal stability. Han et al. [195] first subjected MWCNTs to acid oxidation and purified styrene. Subsequently, they prepared PS@MWCNTs composite materials based on a microemulsion polymerization method and a hot pressing process. Additionally, PS/MWCNTs composite materials were obtained through melt blending and mechanical mixing. Compared to the latter, PS@MWCNTs composite materials exhibit higher thermal conductivity and tensile strength, as well as lower sheet resistance. When the CNTs loading is 10 wt.%, the thermal conductivity is enhanced by 618% compared to pure PS. This enhancement facilitates the expansion of PS applications in thermal conductive composite materials. Furthermore, Zhang et al. [196] used sulfonated MWCNTs (possessing amphiphilicity, hydrophilicity, and oleophilicity) as a surfactant for a water-in-styrene emulsion, maintaining the styrene emulsion in a stable and uniformly dispersed state. CNTs/PS composite materials were manufactured through Pickering emulsion polymerization, and the study indicates that the thermal stability of PS can be significantly improved with a low loading of sulfonated CNTs.

### 4.5. Other Performance and Applications

**Figure 8:**
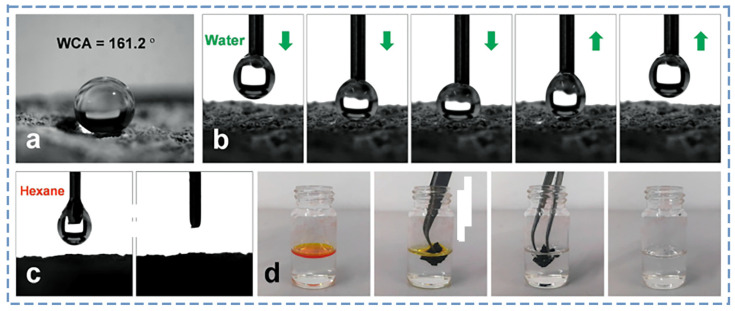
(**a**): Water contact angle test of polystyrene/carbon nanotubes foam (PCF). (**b**,**c**): Water repellency and oil absorption tests of PCF. (**d**): Delective oil absorption from the surface of water using PCF. Reproduced with permission [199]. Copyright 2021, Elsevier Ltd.

Polymer composites with high dielectric constants can be used in various fields such as piezoelectricity, capacitors, and electrical terminals. However, most polymer materials have relatively low dielectric constants. Wu et al. [197] successfully prepared TiO_2_ nanorod-modified MWCNTs (TD-CNTs) through self-assembly of TiO_2_ on the surface of CNTs under high-temperature conditions. Introducing TD-CNTs into PS significantly increased its dielectric constant (up to 13.7 times that of pure PS) with low electrical loss. Moreover, the dielectric properties of TD-CNTs/PS composite materials could be adjusted by changing the content of TiO_2_ nanorods and the length of CNTs. The prepared composite material has potential applications in capacitor materials. CNTs/PS composite materials can also be used for drug delivery. Gul et al. [198] constructed coarse-grained models of PS, carboxyl-functionalized PS, and covalently functionalized CNTs. They used molecular dynamics simulations to study the interaction between lipid bilayers and PS-functionalized CNTs. The simulation revealed that PS-functionalized CNTs could diffuse into the membrane without damaging it, indicating the potential application of PS-functionalized CNTs as drug carriers. Additionally, the simulation suggested that drug loading efficiency could be improved by adjusting the grafting density of PS and the length of the polymer chains. Due to PS’s low surface energy and CNTs’ hydrophobic nature, Shan et al. [199] proposed a two-step method to prepare superhydrophobic and superoleophilic three-dimensional porous PCF for oil–water separation (as shown in Figure 8). The preparation process involved the mixing of CNTs, PS, and the pore-forming agent cinnamic acid (CA), the phase transition of PS in the mixed solution, and the removal of CA. CNTs provided the PS foam with a nanoscale rough structure. The prepared superhydrophobic and superoleophilic PCF can be used for the treatment of chemical liquids and oil spills, and the PS used in the preparation is recycled waste, aligning with green environmental principles.

Nanotopological structures and the morphology and size of nanomaterials can affect the performance of neural tissue interface tools. PS/CNTs composite materials can also be applied to biomedical devices. Calaresu et al. [200] successfully prepared PS/CNTs nanowires (NPs) composite materials with high aspect ratios and high density through a combination of solution blending, flat hot pressing, and nanoimprint lithography. The prepared PS-CNT-NPs demonstrated interesting biomedical applications, including effective electrical stimulation of neural tissues, the ability to maintain the physiological activity of neural tissues, and the advantage of inducing low glial proliferation. Polymer composite materials with conductive properties have advantages such as lightweight and low cost in manufacturing steam sensors, capable of detecting high concentrations of steam leaks. Li et al. [201] used a melt blending method to prepare MWCNTs/polylactic acid/PS conductive composite materials for steam leak detectors. The authors aimed to study the influence of thermal annealing on its steam sensing characteristics, and the results showed that while thermal annealing reduced the relative resistance change, it increased the steam tolerance of the sensor, providing better sensing reversibility. MWCNTs have a large surface area, and the spaces inside and between layers provide numerous adsorption sites for gases [202]. Wu et al. [203] used electrospinning to prepare MWCNTs/PS composite material gas separation membranes. The application of an electric field improved the dispersion of CNTs and allowed for vertical orientation. Compared to composite membranes prepared without an electric field, the electrospun membranes showed a more significant improvement in oxygen permeation, although there was no significant difference in nitrogen permeability.

## 5. Conclusions and Perspectives

### 5.1. Conclusions

In this review, we focused on two primary aspects crucial to advancing the understanding and utilization of CNT/PS composite materials. Firstly, we presented an overview of diverse surface modification methods for CNTs in PS, encompassing both covalent grafting and non-covalent modifications utilizing PS-related polymers, along with alternative surface modification approaches. This comprehensive exploration is imperative to achieve optimal compatibility between CNTs and PS, ensuring uniform and stable dispersion in the polymer matrix. The intricate surface modification of CNTs plays a pivotal role in the preparation of high-performance CNTs/PS nanocomposite materials. Secondly, we delved into the multifaceted properties of CNTs/PS composite materials, including mechanical performance, electrical conductivity, electromagnetic shielding, thermal conductivity, thermal stability, and other significant attributes. The incorporation of CNTs into the PS matrix has proven instrumental in enhancing various material properties, underscoring the importance of surface modification in achieving superior performance. This success has significantly broadened the application spectrum of PS from conventional domains like packaging and construction to cutting-edge fields such as conductivity, thermal conductivity, and electromagnetic interference shielding.

### 5.2. Perspectives

The advancement and refinement of CNTs/PS composite material properties underscore the efficacy of CNTs’ surface modification methods, particularly when employing intrinsic PS or PS-related polymers for both covalent and non-covalent modifications of CNTs. It is reasonable to anticipate that modifying CNTs with polymers akin to or associated with the matrix, and subsequently incorporating them into the polymer matrix, can yield composite material properties surpassing those achieved with non-matrix polymers modified CNTs. The primary objective of these methods is not only to elucidate the surface modification of CNTs in PS and its consequential impact on material properties but, more crucially, to provide a framework for recognizing the approach. Through the judicious utilization of filler and matrix properties, optimal surface modification methods for CNTs in the target polymer can be tailored, maximizing the intrinsic polymer properties. The surface modification methodologies for CNTs in PS can be extrapolated to other carbon materials (including CNTs, graphene, and carbon black) in polymers containing aromatic rings (such as PS, polybutadiene rubber, bisphenol A epoxy resin, and styrene ethylene butylene styrene). Furthermore, this approach can be analogously applied to various nanofillers and polymers.

While the surface modification of CNTs in PS has witnessed diverse methodologies, common challenges persist, necessitating improvements to achieve surface modification that is simple, efficient, environmentally friendly, and applicable in practical scenarios. For covalent grafting surface modification using PS-related polymers, challenges include complex procedures, high reaction requirements, extended modification cycles, elevated costs, involvement of numerous chemical reagents, and difficulties in achieving large-scale surface modification of CNTs. Additionally, similar to most covalent modifications of CNTs, grafting modifications of PS or its related polymers onto CNTs may compromise their intrinsic characteristics, potentially adversely affecting the performance of CNTs and their nanocomposites. In contrast, non-covalent modification using PS-related polymers, while associated with fewer challenges compared to covalent grafting, and more conducive to large-scale industrial production, presents the drawback of weaker interactions at the polymer matrix interface. Moreover, the interface interactions in CNTs/polymer composites are closely linked to various properties, especially mechanical performance. Therefore, when choosing a method for CNTs surface modification, a delicate balance must be struck among modification conditions, cost-effectiveness, interface interaction strength, and overall material performance.

In the study of mechanical properties, electrical conductivity, and electromagnetic shielding properties of CNTs/PS composite materials, several avenues warrant exploration to fully unlock the potential of CNTs/PS or polymer nanocomposites.

(1) Orientation Control for Anisotropy: Tensile and melt shear processes can control the orientation of CNTs, imparting anisotropy to CNTs/polymer nanocomposites. However, a singular orientation of CNTs can reduce the formation of conductive pathways, potentially hindering conductivity. Improving preparation methods, such as utilizing stacking or interweaving techniques to make CNT orientation more complex and unique, may unlock more intriguing material properties.
(2) Dispersion Enhancement Strategies: The dispersion of CNTs is a critical issue in the preparation process of CNTs/PS or polymer composite materials. Exploring methods such as electrospinning could offer solutions to address dispersion challenges, providing a convenient approach for the preparation of CNTs–polymer composite materials.
(3) Enhancing Interface Microstructure: The interface microstructure and percolation network formed between CNTs and the matrix in CNTs/PS composite materials are currently limited. Expanding and optimizing these aspects could unleash the full potential of the mechanical and electrical properties of CNTs/PS or polymer composite materials. CNTs can synergistically form unique multilevel microstructures and conductive networks with other materials, enhancing overall performance.
(4) Characterization of Interface Interactions: Understanding the interface characteristics of CNTs/PS composite materials and achieving a customized interface are crucial for manufacturing high-performance CNTs composite materials. Characterizing interface interactions, especially changes in the interaction between CNTs and the polymer matrix before and after CNTs’ modification, is essential. Direct measurement methods using equipment pose challenges, but advancements in computer technology and the maturity of molecular dynamics simulation make it more convenient to study the influence of CNTs’ surface modification on interface interactions in polymer composite materials by constructing molecular dynamics models.

## Figures and Tables

**Table 1:** Types of CNT–polymer interactions and energies.

Type	Interaction Energy (kcal/mol)
Van der Waals	~0.1–1
π–π stacking [41]	~10
CH–π/NH–π [42]	~1–10
Hydrogen bond	~2–30

**Table 2:** Surface modification methods of CNTs in PS.

Methods	Characterizations	Mechanisms	Advantages	Disadvantages
Covalent grafting of PS-related polymers	PS-related polymers are directly or indirectly connected to CNTs through chemical bonds.	Chemical reaction, partial change in carbon atoms from sp^2^ hybridization to sp^3^.	Strong bonding between the surface modification agent; high compatibility with the PS matrix.	The preparation process is complex and costly, involves a significant amount of chemical reagents, and results in the destruction of the inherent characteristics of CNTs.
Non-covalent modification of PS-related polymers	PS-related polymers encapsulate/wrap around the surface of CNTs through physical interactions.	Van der Waals forces, electrostatic interactions, π–π stacking, hydrophobic interactions, physical adsorption, steric hindrance.	Intact sp^2^ carbon atomic structure and the properties of CNTs are maintained; high compatibility with PS.	The interaction between CNTs and polymers is relatively weak.
Other methods	The modification process does not involve PS or PS-related polymers.	Chemical reaction, partial change in carbon atoms from sp^2^ hybridization to sp^3^, van der Waals forces, amide–π, π–π, π–cation interactions, physical adsorption, steric hindrance.	The method is highly selective and has a wide range of applicability, most of them are simple and easy to operate.	Compared to PS-related polymers, the compatibility with PS for modifying CNTs is lower.

**Table 3:** Effects of different CNTs’ functionalization methods on their dispersion and polymer composite properties (A: Atom transfer radical polymerization; B: Other covalent grafting methods; C: Non-covalent modification using PS-related polymers; D: Other methods.).

Method	Types of CNTs	Dispersion Improvement	Property Improvement	Ref.
A	MWCNTs	Disperses well in CH_2_Cl_2_, Tetrahydrofuran (THF) and toluene	-	[92]
A	MWCNTs	Disperses well in THF and benzene	-	[93]
A	SWCNTs	-	Increased mechanical properties	[94]
A	SWCNTs	Disperses well in THF	-	[95]
A	SWCNTs	Disperses well in THF	Increased *T_g_* and conductivity	[96]
A	MWCNTs	Disperses well in solution	Increased tensile strength	[97]
A	MWCNTs	Disperses well in solution	Improved *T_g_* and thermal stability	[98]
A	MWCNTs	Disperses well in PS	Improved thermal stability	[99]
A	MWCNTs	Disperses well in toluene and xylene	Increased *T_g_* and conductivity	[101]
A	MWCNTs	-	Increased elastic modulus	[102]
A	MWCNTs	Dispersed well in polymers	Good compatibility	[103]
A	MWCNTs	Disperses well in PS	Surface resistance and percolation threshold are reduced, and compatibility is good.	[104]
B	MWCNTs	Improved dispersion	Improved impact and tensile strength, as well as thermal stability, good compatibility	[105]
B	SWCNTs	Dispersed well in THF, CHCl_3_, and CH_2_Cl_2_	-	[107]
C	SWCNTs	Disperses well in PS solution	Low current percolation threshold (0.095 wt.%)	[4]
C	MWCNTs	Evenly dispersed and stable in solution	-	[109]
C	MWCNTs	Disperses well in PS	-	[110]
C	MWCNTs	Disperses well in organic solvents	-	[111]
C	SWCNTs	Disperses well in PS solution	-	[112]
D	MWCNTs	Improved dispersion	Improved conductivity (percolation threshold is 0.05–0.08 wt.%) and electromagnetic shielding performance	[1]
D	MWCNTs	Disperses well in PS	Enhanced photoelectric and rheological properties	[121]
D	MWCNTs	Disperses well in PS	Low current and rheological thresholds	[122]
D	MWCNTs	Disperses well in PS	Improved electrical conductivity and thermal stability	[123]
D	MWCNTs	Disperses well in PS	Increased conductivity, tensile strength, and modulus	[125]
D	MWCNTs	Disperses well in PS	Improved conductivity (current percolation threshold 0.5–1 wt.%) and thermal stability	[126]
D	CNTs	Improved dispersion	Increased conductivity (current percolation threshold is 1.44 wt.%)	[130]
D	MWCNTs	Disperses well in PS	Increased tensile strength and elongation at break	[131]
D	MWCNTs	Improved dispersion	Good electrical conductivity (current percolation threshold is 0.05 wt.%)	[132]

**Table 4:** Electromagnetic shielding properties of CNTs/PS composites.

Types of CNTs	Method	CNTs Content	Percolation Threshold	Frequency (GHz)	EMI SE	Ref.
MWCNTs	Build a separation structure	2.0 wt.%	0.009 vol%	8.2–12.4	55.7 dB/mm	[173]
CNTs	Foaming and sintering	0.046 vol%	0.0014 vol%	12.4	211.5 dB cm^3^ g^−1^	[174]
MWCNTs	Injection molding Compression molding	5.0 wt.%	-	8.2–12.4	8.05–11.46 dB 17.2 dB	[181]
MWCNTs	Electrospinning	7.5 wt.%	0.45 vol%	8.2–12.4	32 dB	[183]
MWCNTs	Foaming	7.0 wt.%	-	8.2–12.4	≈20 dB	[184]

## Data Availability

Not applicable.

## Abbreviations

AIBN	Azobisisobutyronitrile
ATRP	Atom transfer radical polymerization
AFM	Atomic force microscopy
CA	Cinnamic acid
CNT	Carbon nanotube
CPC	Conductive polymer composite
CPN	Electrically conductive polymer nanocomposite
CTAB	Cetyltrimethylammonium bromide
DMF	Dimethylformamide
DWCNT	Double-wall carbon nanotube
EG	Expanded graphite
EMI	Electromagnetic interference
EVA	Ethylene–vinyl acetate
FTIR	Fourier transform infrared spectroscopy
GNS	Graphene nanosheet
MWCNT	Multi-wall carbon nanotube
NaDDBS	Sodium dodecylbenzenesulfonate
P3HT	Polythiophene
PA6	Polyamide-6
PCF	Polystyrene/carbon nanotubes foam
PDA	Polydopamine
PEDOT:PSS	Poly(3,4-ethylenedioxythiophene): poly(styrene sulfonate)
PMMA	Poly(methyl methacrylate
PP	Polyphenylene
PPO	Polyphenylene ether
PS	Polystyrene
PyPS	Pyrene-terminated polystyrene
SDS	Sodium dodecyl sulfate
SE	Shielding effectiveness
St	Styrene
SWCNT/SWCNT	Single-wall carbon nanotubes
TEM	Transmission electron microscope
THF	Tetrahydrofuran
TEMPO	2,2,6,6-tetramethylpiperidine-1-oxy
TMSPMA	3-(trimethoxysilyl)propyl methacrylate
UVO	Ultraviolet ozone
*T_g_*	Glass transition temperature
NaN_3_	Sodium azide
